# The Neural Basis of Responsibility Attribution in Decision-Making

**DOI:** 10.1371/journal.pone.0080389

**Published:** 2013-11-05

**Authors:** Peng Li, Yue Shen, Xue Sui, Changming Chen, Tingyong Feng, Hong Li, Clay Holroyd

**Affiliations:** 1 Research Center of Psychological Development and Education, Liaoning Normal University, Dalian, China; 2 School of Psychology, Liaoning Normal University, Dalian, China; 3 Institute of Neuroinformatics, Dalian University of Technology, Dalian, China; 4 School of Psychology, Southwest University, Chongqing, China; 5 Department of Psychology, University of Victoria, Victoria, British Columbia, Canada; George Mason University / Krasnow Institute for Advanced Study, United States of America

## Abstract

Social responsibility links personal behavior with societal expectations and plays a key role in affecting an agent’s emotional state following a decision. However, the neural basis of responsibility attribution remains unclear. In two previous event-related brain potential (ERP) studies we found that personal responsibility modulated outcome evaluation in gambling tasks. Here we conducted a functional magnetic resonance imaging (fMRI) study to identify particular brain regions that mediate responsibility attribution. In a context involving team cooperation, participants completed a task with their teammates and on each trial received feedback about team success and individual success sequentially. We found that brain activity differed between conditions involving team success vs. team failure. Further, different brain regions were associated with reinforcement of behavior by social praise vs. monetary reward. Specifically, right temporoparietal junction (RTPJ) was associated with social pride whereas dorsal striatum and dorsal anterior cingulate cortex (ACC) were related to reinforcement of behaviors leading to personal gain. The present study provides evidence that the RTPJ is an important region for determining whether self-generated behaviors are deserving of praise in a social context.

## Introduction

Actions are guided by a sense of personal responsibility that follows moral principles [1]. Responsibility attribution -- determining who is responsible for what outcomes -- underlies this process and is fundamental to the allocation of social resources. For example, people who feel guilty for bad behavior might alter their behavior in the future. Recently, neuroscientists have become greatly interested in exploring the neural basis of social cognition and moral judgement [2,3]. However, the neural basis of social responsibility attribution has yet to be well explored. 

Schlenker and colleagues defined responsibility as a psychological adhesive that connects an actor to an event in accord with behavioral prescriptions [4]. They constructed a triangle model of responsibility containing three elements: *prescriptions*, *events* and *identity*. Prescriptions refer to general codes or rules for conduct, that is, the rules that the actor should follow in any particular situation (e.g., “students should not make noise in class” versus “construction workers are allowed to make noise”). Events constitute the actions themselves together with their consequences (e.g., “Making noise in class is disruptive to the professor’s lecture” versus “asking good questions in class can be helpful to everyone”) and identity refers to the actor’s roles and commitments (e.g., “The person who made noise is a student enrolled in the class” versus “The person who made noise is a construction worker outside”). Schlenker and colleagues proposed that the sense of responsibility (responsibility sense) depends on the strengths of three linkages between each two of the three elements and, prior to performing an action, affects the actor’s determination to achieve the associated goal. 

Social psychologists since Heider (1958) have investigated how people attribute responsibility [5]. Typically, attributions are coloured by a *self-serving bias*: individuals attribute positive outcomes to their own actions and explain away negative outcomes to external factors or other people [6,7]. The self-serving bias appears to enhance self-esteem [4]. Weiner (1985) suggested a theoretical framework for understanding attribution in which “cognitions of increasing complexity” sequentially induce corresponding emotional processes [8]. Specifically, he suggested that following an outcome to a behavior, people experience emotions based on the success or failure of the outcome, such as happiness or sadness, that are independent of the source of the behavior. Immediately after this, they experience a different set of emotions based on their attribution of the source of the behavior, such as guilt and pride. To be clear, here we define *responsibility sense* as an actor’s perceived degree of responsibility before and during an action and *responsibility attribution* as their feelings of guilt or pride subsequent to the action. 

 Many studies have demonstrated that responsibility sense dramatically modulates emotional states following decisions, for example, feelings of regret or disappointment [9]. We have also confirmed this finding in previous ERP studies. In one study, responsibility sense was manipulated according to whether participants worked as individuals within a group or on behalf of the whole group [10]. Participants reported higher responsibility sense when working individually than with other teammates. Further, neural activity associated with outcome evaluation, as indexed by a component of the event-related brain potential (ERP) called the feedback error related negativity (fERN), was greater in a high- relative to a low-responsibility condition. In addition, fERN amplitude was significantly correlated with subjective ratings of responsibility but not with subjective ratings of happiness. These results were supported by another study that investigated how participants’ responsibility sense was affected by their subjective sense of control over a gambling task [11]. 

The reinforcement learning theory of the fERN suggests that this outcome evaluation process is mediated by brain areas that support reinforcement learning, such as the basal ganglia, ACC, and other motor-related neural areas [12]. An interesting question concerns whether this neural network for reinforcement learning is in fact modulated by responsibility sense as suggested by the ERP studies. Several fMRI experiments have demonstrated that responsibility sense affects the BOLD signal of brain regions involved in reward processing as observed in a regret-based decision making task. For example, in a “wheel of fortune” gambling task, Coricelli and colleagues found that the sense of personal responsibility influenced outcome evaluation by showing that feedback in a condition involving a sense of agency aroused stronger activation in ventral striatum than feedback in a condition that did not involve a sense of agency [13]. Subsequent studies also revealed that reward regions, especially in the striatum, that responded differentially to win and loss were also modulated by personal responsibility [14,15]. Additionally, a recent study demonstrated an enhanced amygdale response to regret-related outcomes when these outcomes were associated with individual responsibility [16]. These studies provided strong evidence that responsibility sense modulates reward processing in decision making tasks. However, because reward-related factors were confounded with responsibility in these experiments, responsibility attribution could not be examined. 

In contrast to the many studies on responsibility sense, only a few studies have examined the neural basis of responsibility attribution. Blackwood et al. conducted an fMRI study to explore the neural basis of responsibility attribution and the self-serving bias in attributional decision tasks [17]. Participants were asked to read and vividly imagine specific events as happening to them, and then to attribute the cause of those events. For example, participants were asked to indicate whether the sentence “A friend bought you a present” was about them, their friend, or the situation itself. They found that the dorsal striatal activity was modulated by a self-serving bias (in which relatively more positive than negative events were self-attributed) whereas brain areas subserving action-simulation—including bilateral premotor cortex and the cerebellum--were modulated by attributions of self-responsibility (in which relatively more negative than positive events were self-attributed). This study thus revealed that different parts of the brain subserve the self-serving bias and self-responsibility. Note that this decision making task depended on mental simulation of observed (as opposed to self-generated) behaviors. More recently, Young and colleagues asked participants to judge the behaviors of the protagonists in various stories. They predicted that brain regions related to theory of mind (ToM) would also be involved in moral evaluation and found that RTPJ was selectively activated by negative moral judgements [18-20].

 All of the above studies have investigated responsibility attribution and moral judgement from the perspective of an independent observer. However, actors vs. observers can attribute social events to different causes because of cognitive and motivational biases [21,22]. Therefore, neural processes underlying responsibility attribution may differ for self-generated vs. externally-generated behaviors. 

**Figure 1:**
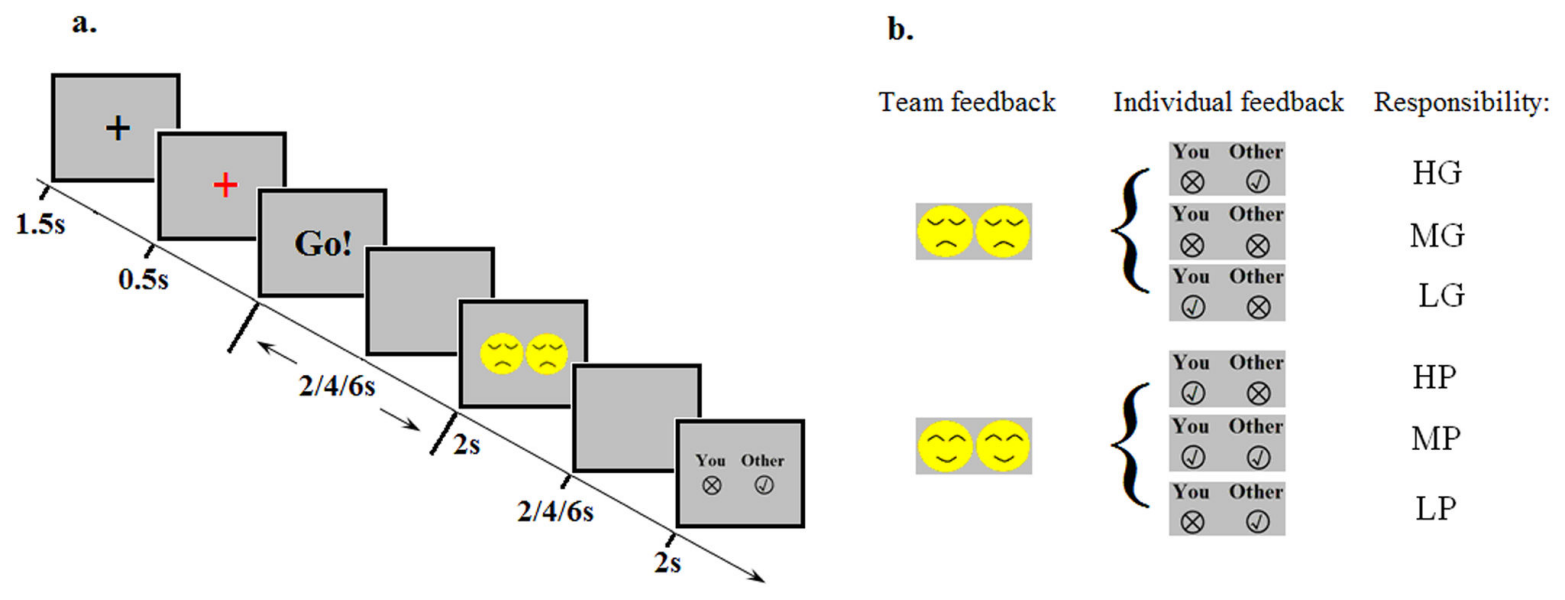
Illustration of the experimental task (a) and experimental design matrix (b). See text for details.

Here we investigated responsibility attribution in a decision making task that separated cognitive processes involved with attribution from processes involved in outcome evaluation. Specifically, in the present study we conducted an fMRI experiment that required participants to cooperate in the task as part of a team and that sequentially presented team outcomes (*team feedback*) followed by individual outcomes (*individual feedback*). Our study differs from previous research in two important respects. First, we focused on self-generated behaviors rather than observed behaviors. In other words, responsibility attribution in the present experiment was from a first-person view rather than from a third-person view. Second, we focused on responsibility attribution to feedback while controlling for the effect of the feedback on reward processing. This allowed us to dissociate responsibility attribution from reward processing of monetary outcomes, which would not have been possible if information related to responsibility and reward were delivered simultaneously. According to Weiner’ theoretical framework, in the present study when participants receive individual performance feedback they should attribute the source of the outcome differently depending on the context, namely, whether the team won or lost [8]. To be clear, we call the attribution-dependent emotion *guilt* when the team outcome was negative and *pride* when the team outcome was positive (Figure 1). 

A previous study demonstrated that the “self system” is engaged in responsibility processing and that social context influences this process [4]. It has also been shown that difficulties in attributing responsibility and in experiencing pride were observed in autism, which is associated with ToM impairments [23,24]. Therefore, we predicted that neural mechanisms implementing responsibility attribution might overlap with brain regions subserving *self-referential processes* and ToM, such as MFC, TPJ and precenues [3,25-29]. Moreover, because individual feedback in our study also provided information necessary for error monitoring, we predicted that the reinforcement learning system, including parts of the basal ganglia, ACC and other motor areas, might also be activated in this task. 

## Methods

### Participants

Eighteen healthy volunteers (6 males; mean age=21.1 years, Standard Deviation, SD=1.1 years) participated in the current study. All of the subjects were right-handed with normal or corrected-to-normal vision. Three of them were excluded from data analysis because of head movement during scanning (one participant) or because they were categorized as an outlier because the number of error trials was more than two SDs from the mean (one participants)[30]. An additional participant was categorized as an outlier because self-reports of pride were more than two SDs below the mean. Thus fifteen subjects were included in the final analysis. This study has been approved by the IRB at Southwest University. We had obtained appropriate ethics committee approval for the research reported, and all subjects gave written informed consent in our experiment. 

### Stimuli and task design

This experiment consisted of two sessions, a pre-scan behavioral test session and an fMRI scan session the following day. For session one, participants were required to engage in a standard time estimation task in which the time window changed according to participants’ accuracy; the task is designed such that the feedback depends on performance and yet results in 50% correct feedback [31]. Participants were told that the aim of this session was to test their time estimation ability and that they would be assigned to a two-person team with another stranger who had the same level of time estimation ability. The following day, they finished a team cooperation task with their partners (who were actually confederates of the investigator) in the fMRI experiment. Note that the real participants were always assigned to play the game in the fMRI scanner. 

 For the fMRI session, at the beginning of each trial of the time estimation task a black fixation cross appeared on a gray background (1500 ms) and then turned red (500 ms) (Figure 1a), serving as a warning stimulus. Afterwards, the word “Go!” appeared on the screen (2000 ms maximum). The participants were told to press a button when they believed that 1000 ms had elapsed since stimulus onset, after which the “Go!” sign disappeared, resulting in a blank screen of random duration; the total duration of the “Go!” stimulus and the blank screen was fixed at random, integral multiples of the TR (i.e., 2000 ms, 4000 ms, and 6000 ms). Then, a “team feedback” stimulus appeared (2000 ms), indicating whether the team won or lost, represented by a smiling face and crying face, respectively, followed by another blank screen with random duration (2000 ms, 4000 ms, 6000 ms). Finally, “individual feedback” appeared (2000 ms), whereby information about the participant’s own performance and his/her partner’s performance was displayed under the words “You” and “Other”, respectively. A cross in a circle indicated poor performance and a check mark in a circle indicated good performance. Participants were told that team success depended on the average performance of their own reaction time (RT_self_) and their partner’s RT (RT_other_) according to the following rule: RT for team (RT_team_)=(|RT_self_-1s|+ |RT_other_-1s|)/2. Thus, if the average RT was within the time window from 0 to 100 ms then the team won, otherwise, they lost. In fact, unknown to participants the team feedback was pseudo-randomly generated such that it indicated success on half of the trials and failure on the other half. However, for trials with RTs longer than 1800 ms or shorter than 400 ms, team feedback was always negative and individual feedback always indicated guilt, so as to make the feedback seem believable for extreme errors. All stimuli appeared in the center of the screen and were approximately 5 degrees of angle. 

 According to our hypothesis, participants would attribute responsibility differently in accordance with team and individual feedback. The two feedback conditions together describe six conditions: high guilt (HG: the team failed because of the participant’s own bad performance), medium guilt (MG: the team failed because both members performed badly), low guilt (LG: the team failed because of the partner’s bad performance), high pride (HP: the team succeeded because of the participant’s own good performance), medium pride (MP: the team succeeded because both players performed well), and low pride (LP: the team succeeded because of the partner’s good performance) (see Figure 1b). The experiment was divided into four runs. In total, there were 108 trials for each condition of team feedback (team won vs. lost) and 36 trials for each of the six combinations of individual and team feedback.

 After participants finished the experiment they were asked to finish a questionnaire consisting of three parts. In the first part, they had to complete a 9-point questionnaire to rate their degree of happiness when they faced two different situations of team feedback and six situations of individual feedback. Also, they were required to rate their feeling of responsibility (pride and guilt) in relation to different types of individual feedback. “1” indicated very low pride or guilt when the question was related to responsibility attribution and indicated very unhappy when the question related to happiness. Conversely, “9” indicated very high pride/guilt and very happy, respectively. In the second part of the questionnaire they were presented with the following forced-choice question: “Which is more important for you, the success of your team or your own performance?” The third part contains eight questions from the subscale “dutifulness” of big five questionnaire. Since no significant result was found with regard to the score of “dutifulness”, the data of third part will no longer be discussed in the rest of this paper.

### Image acquisition

Blood oxygen level dependent (BOLD) signals were measured using a 3.0 T Siemens MAGNETOM Trio scanner (Allegra; Erlangen, Germany) with a 12-channel head coil. Functional MRI data were acquired in four separate runs using a T 2-sensitive gradient echo planar imaging sequence covering the whole-brain (32 slices, slice-thickness 3.0 mm, Repetition Time (TR)= 2000 ms; Echo Time (TE)=29 ms, ﬁeld of view (FoV): 220 mm^2^, matrix size: 64 × 64. An anatomical 3D dataset consisting of 176 slices was acquired between the second run and the third run (MDEFT sequence (Deichmann et al., 2004); TR= 1900 ms; TE =2.52 ms; Flip Angle=9°, voxel dimensions=1×1×1 mm^3^; FoV=250 mm^2^).

### Behavioral data analysis

Unlike the standard 1 s time-estimation task [31], in the fMRI component of the present study all of the feedback stimuli were presented pseudo-randomly. Therefore, participant accuracy was not meaningful in this experiment. Nevertheless, participants could modify their behavior on a given trial based on feedback received on previous trials. For this reason, we calculated the absolute value of difference in RT between consecutive trials (△RT) as a measure of error correction. In addition, correlation analyses were conducted among responsibility scores, happiness scores and △RT values. All the r-values reported here reflect Pearson correlation values. 

### fMRI data analysis

Imaging data were analysed using BrainVoyager QX (Brain Innovation, Maastricht, the Netherlands). Functional data were preprocessed to correct for slice scan time differences (using sinc interpolation), 3D motion artifacts (Trileaner sinc interpolation), linear drifts, and low-frequency non-linear drifts (high pass filter less than 2 cycles/time course). Functional data were then co-registered with the anatomical volume and transferred into standard stereotaxic space using Talairach normalization [32] and spatially smoothed with a 4 mm full width at half maximum Gaussian kernel.

 The statistical analyses were carried out using a voxel-wise General Linear Model (GLM) at the single subject level, based on design matrices that included the estimated 3D motion parameters obtained during pre-processing as well as predictors for all relevant task conditions (two events of team feedback, six events of individual feedback and the “Go!” stimulus). The reported group analyses were conducted using a random effects model. Note that only the time periods associated with team feedback and individual feedback were of interest. The analyzed time window of each event was 2s. And the box-car regressor of feedback stimulus was calculated throughout the entire duration of feedback presentation. The time of box-car regressors of all conditions were the same. The two conditions of team feedback (team loss and team gain) were submitted to a T-test, and the three pride conditions and three guilt conditions were submitted to ANCOVA analysis in Brain Voyager separately. The statistical group maps representing significant results were corrected for multiple comparisons using the false-discovery rate (q <.05, FDR) with 10 continuous voxels [33]. 

 As an exploratory analysis, regions of interest (ROIs) were created based on the significant clusters of activation identified in voxelwise analyses of group level for the main effects of pride and guilt attribution (see results). The ROI was defined for each subject based on the peak coordinates of the results from the group level. Then, ROI analyses were performed by extracting parameter estimates (betas) from the GLM model and averaging across all voxels in the cluster for each subject. These beta values were further subjected to correlation analyses between the BOLD and behavioral data. For the pride and guilt conditions separately, correlations were performed across the three sub-conditions (high, medium, low) between the BOLD signal and △RT. We also computed differences in behavioral data (separately for RT, happiness scores and responsibility scores) between pairs of individual feedback conditions (e.g., HP and MP), and correlated these values with the corresponding differences in the BOLD signal for each of the ROIs, separately. 

## Results

### Behavioral data

**Figure 2:**
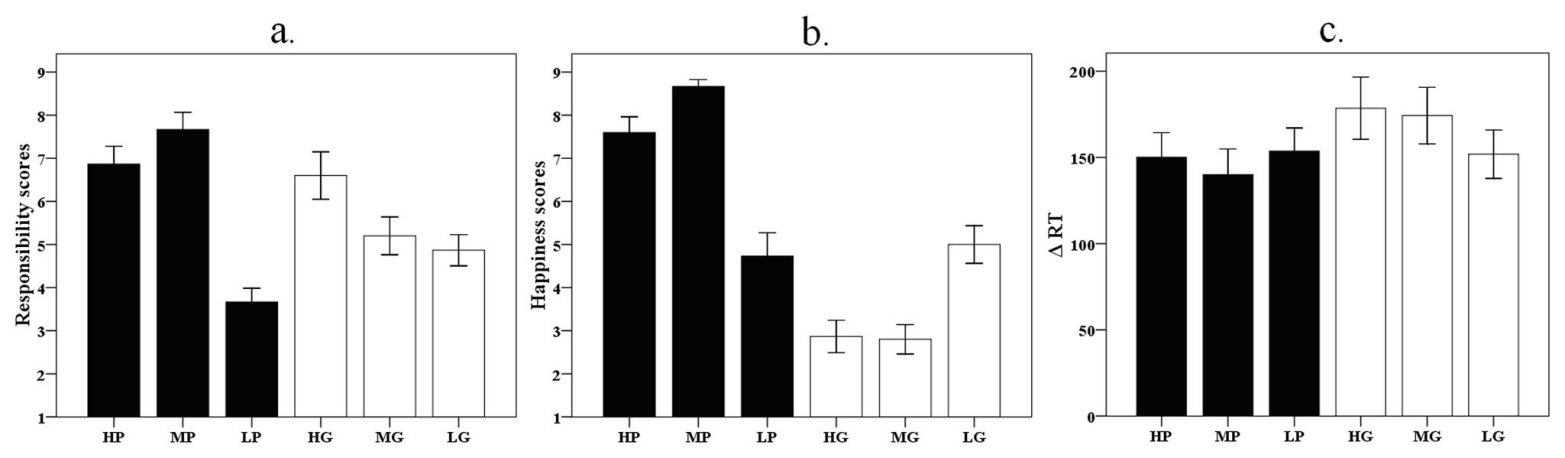
Behavioral data: the mean value of subjective rating scores of responsibility sense (pride and guilt, panel a), happiness (panel b) and △ RT (panel c). Error bars indicate +1 sem.

Because the △ RT values are related to performance monitoring and error correction, these △ RT data were entered into a three (responsibility level: high, middle, low) by two (valence: guilt and pride) repeated measure analyses of variance (ANOVA). The results revealed a main effect of valence, F (1, 14) =16.23, *p* =.001 (Figure 2). The △ RT was larger after the team lost feedback (i.e., guilt condition: 168±57 ms) than that after team won feedback (i.e., pride condition: 148±49 ms), indicating that participant changed their time estimate more following monetary losses than monetary gains. There was no main effect of responsibility, F (2, 28) <1, *p* =.47, and no significant interaction between valence and responsibility, F (2, 28) =3.46, *p* >.05. 

Given that people could attribute the source of the outcome differently following positive vs. negative team outcomes—that is, they could show a self-serving bias [34,35] --the BOLD signal of responsibility attribution and subjective rating scores were analyzed separately for the pride and guilt conditions (Figure 2a). First the subjective rating scores of pride in the pride conditions were submitted to a one-factor repeated measures ANOVA with three levels (HP, MP and LP). The ANOVA revealed a main effect of pride, F (2, 28) =36.94, *p* <.001. Follow-up T-tests revealed higher reported pride in the MP condition (7.7± 1.5) compared to the HP condition (6.9±1.6), t(14)=2.26, *p* <.05, and higher reported pride in the HP condition compared to the LP condition (3.7±1.2), t(14)=6.18, *p* <.001. Second, the subjective rating scores of guilt in guilt conditions were submitted to a one factor repeated-measures ANOVA with three levels of guilt (HG, MG, and LG). The main effect of guilt was significant, F (2, 28) =4.31, *p* <.05. Participants felt more guilt in the HG condition (6.6±2.1) compared to the MG condition (5.2±1.7), t(14)=2.77, *p* <.05, and more guilt in the HG condition compared to the LG condition (4.9±1.4), t(14)=2.39, *p* <.05. Guilt was not significantly different between the MG and LG conditions, t(14)=0.61, *p* >.05. 

 Afterwards, the subjective rating scores of happiness in the pride condition were submitted to a one-factor repeated measures ANOVA with three levels of pride (HP, MP and LP), revealing a main effect of happiness, F (2, 20) =45.1, *p* <.001 (Figure 2b). Pairwise comparison revealed significant differences between each level of pride. As illustrated in Figure 2, people reported more happiness in the MP condition (8.7±0.6) than in the HP condition (7.6±1.4), t(14)=3.38, *p* =.005. Happiness scores were also higher in the HP condition than in the LP condition (4.7±2.1), t(14)=7.37, *p* <.001. Likewise, the subjective rating scores of happiness in the guilt condition were submitted to a one-factor repeated measures ANOVA with three levels (HG, MG and LG), revealing a main effect of happiness, F (2, 28) =16.57, *p* <.001. Pairwise comparison showed that participants reported greater happiness in the LG condition (5.0±1.7) than in the HG condition [2.8±1.5, t (14) =4.68, *p* <.001] and than in the MG condition [2.8±1.3, t(14)=4.32, *p* =.001]. No difference was found between the HG condition and the MG condition, *p* >.05. In addition, twelve out of fifteen subjects reported that team success was more important to them than their own success. 

A subsequent correlation analysis revealed that pride was significantly correlated with happiness in the pride condition, r =0.72, *p* <.001. By contrast, guilt was significantly negatively correlated with happiness in the guilt condition, r = -0.30, *p* <.05. No significant correlations were found between the △ RT values and subjective rating of happiness and responsibility in both conditions (all *p* >.05). 

### fMRI data

#### Team feedback

**Figure 3:**
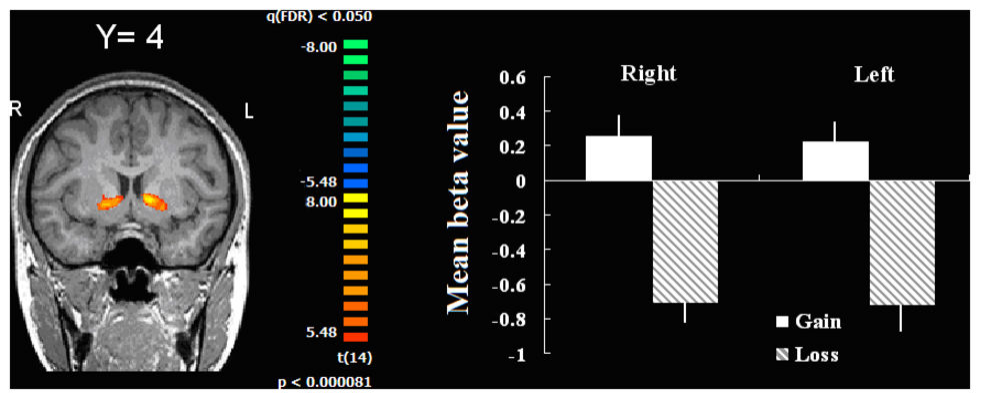
Brain areas showing greater activity for win team feedback than lose team feedback (left pane) and associated BOLD signal changes separately for left and right dorsal striatum (right pane). Error bars indicate standard error.

As indicated in Table 1 and Figure 3, a whole brain random effects analysis comparing the team win condition with the team lose condition revealed activation in the bilateral dorsal striatum only: Positive feedback induced greater activity than did negative feedback, consistent with previous research [36,37]. This result confirmed the validity of the present manipulation. Because the team feedback is not the event of main interest, these results will not be further discussed. 

**Table 1:** Brain areas showing greater activity for win team feedback than lose team feedback.

**Brain area**	**Hemisphere**	**Talairach coordinate**			**Effect size**		**Voxels**
		**X**	**Y**	**Z**	**t-value**	***P*-value**	
Dorsal striatum	Right	15	-1	-5	9.51	0.000001	51
Dorsal striatum	Left	-12	5	1	9.26	0.000001	37

The t-value, P-value and coordinate are from the peak voxel within each region of interest.

#### Individual feedback

**Figure 4:**
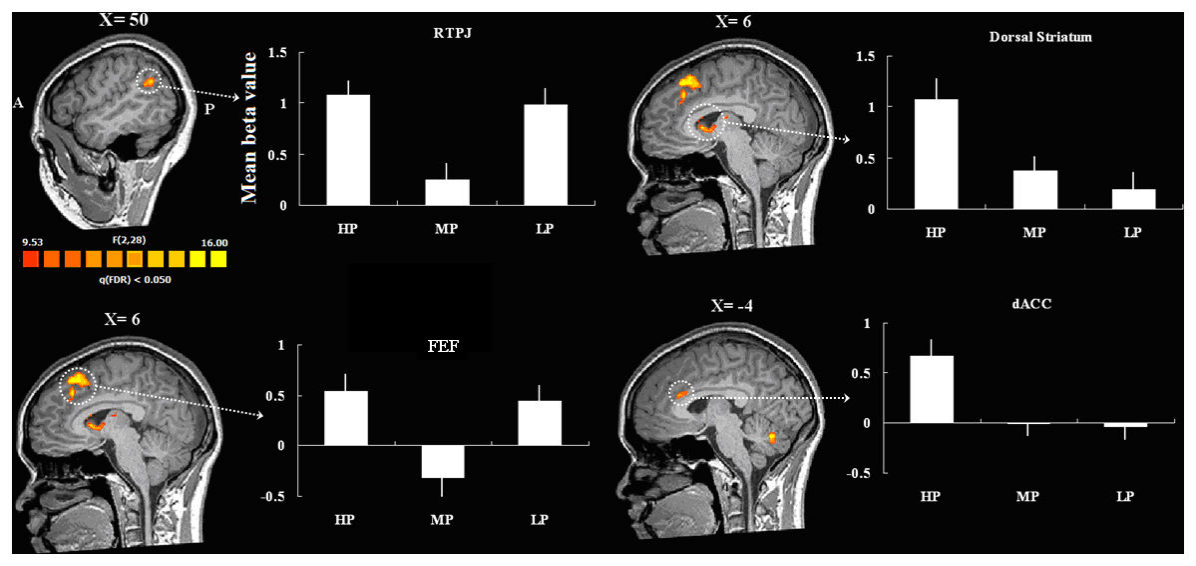
Brain areas sensitive to level of pride in the pride condition and corresponding BOLD signal changes within these regions. Error bars indicate standard error.

A one way ANOVA with three levels (HP, MP, and LP) on the BOLD signal associated with the pride condition revealed several brain regions that were differentially sensitive to the level of pride, including the frontal eye field (FEF), ACC and dorsal striatum, RTPJ, inferior temporal gyrus, occipital cortex, and others (Table 2). We focused our analyses on dorsal striatum and dACC because of their demonstrated involvement in RL [12] and, as exploratory analyses, on the FEF and RTPJ because the BOLD responses in these brain areas were correlated with our behavioral measures. Figure 4 presents statistical maps and mean beta values for these regions: RTPJ, FEF, dorsal striatum and dACC. Post-hoc t-tests revealed different patterns of activation among the different regions, as indicated in Table 2. 

**Table 2:** Brain regions sensitive to levels of pride in the pride condition.

**Groups**	**Brain area**	**Hemisphere**	**Talairach coordinate**			**Effect size**		**Voxels**
			**X**	**Y**	**Z**	**F-value**	***P*-value**	
HP=LP>MP	RTPJ(40)	R	51	-52	28	17.64	0.000011	25
	FEF(8)	R	3	23	46	22.46	0.000002	86
HP>MP=LP	MOG(18)	R	21	-88	-5	30.11	0.000001	134
	Fusiform Gyrus (37)	R	33	-46	-11	27.92	0.000001	47
	Thalamus	R	15	-13	13	15.25	0.000033	12
	Caudate Head	R	6	11	4	16.99	0.000015	30
	Posterior cerebellum (Declive)	L	-6	-64	-20	20.08	0.000004	39
	ACC(24)	L	-3	23	19	15.41	0.000031	11
	Lentiform Nucleus	L	-9	-1	4	17.15	0.000014	12
	PCC (30)	L	-18	-64	10	20.74	0.000003	32
	IOG (18)	L	-27	-88	-8	19.39	0.000005	23
HP>LP>MP	MFG (9)	R	39	26	25	15.99	0.000023	25
	SPL (7)	R	30	-55	43	16.61	0.000018	11
	Precuneus (7)	R	3	-58	43	15.57	0.000028	13
	Posterior cerebellum (Pyramis)	L	-24	-61	-29	18.61	0.000007	42
	Thalamus	L	-21	-22	-2	17.15	0.000014	16
	Fusiform Gyrus (37)	L	-39	-52	-11	25.79	0.000001	117

These regions were separated into three groups based on t-test results of beta values in each condition. “>”means significantly larger; “=”means not significantly different. T-values, p-values and coordinates are associated with the peak voxel within each region of interest. Abbreviation: RTPJ, right Temporoparietal Junction; FEF, frontal eye field; MOG, medial occipital gyrus; PCC, posterior cingulate cortex; IOG, inferior occipital gyrus.

**Figure 5:**
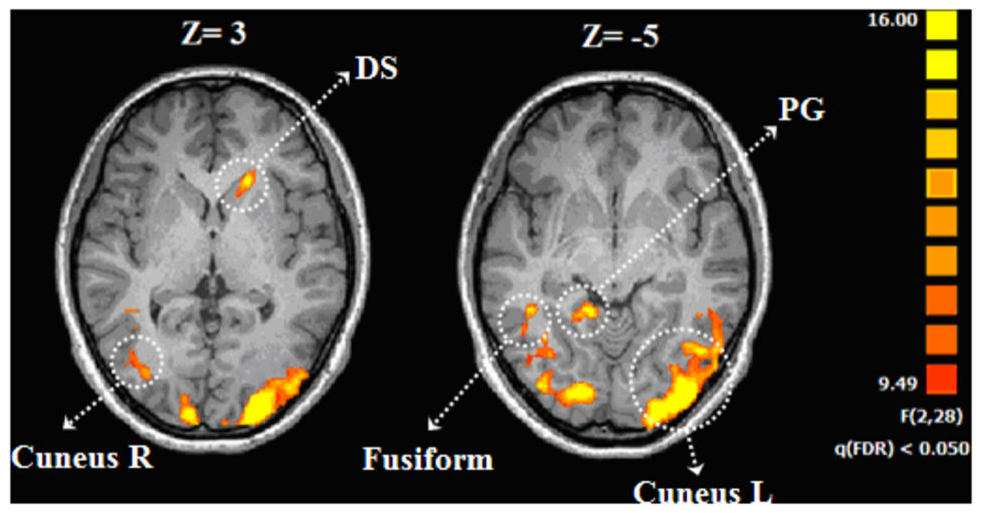
Brain regions associated with the effect of guilt. Error bars indicate standard error. Abbreviation: DS , dorsal striatum ;PG ,parahippocampal gyrus.

 A three-level ANOVA (HG, MG, LG) on the BOLD response associated with the guilt condition revealed only a few clusters sensitive to guilt attribution in a whole brain random analysis, including caudate, bilateral cerebellum and occipital cortex as listed in Table 3 (Figure 5). Associated beta values were correlated with behavioral data across the three guilt conditions (HG, MG, LG). 

**Table 3:** Brain regions associated with the main effect of guilt.

**Groups**	**Brain area**	**Hemisphere**	**Talairach coordinate**			**Effect size**		**Voxels**
			**X**	**Y**	**Z**	**F-value**	***P*-value**	
MG>HG=LG	Caudate Head	L	-18	23	4	24.88	0.000001	21
	MOG (19)	R	30	-76	19	18.05	0.000009	47
MG>HG>LG	Cuneus (17)	R	12	-97	1	31.53	0.000001	10
	Fusiform Gyrus (37)	R	42	-40	-5	16.56	0.000018	11
	Parahippocampal Gyrus (30)	R	15	-40	-5	17.39	0.000012	21
	Cuneus (17)	L	-21	-94	1	54.94	0.000001	670

These regions were separated into two groups according to t-test results on the beta values for each condition. “>”means significantly larger; “=”means not significantly different between. The t-values, p-values and coordinates are from the peak voxel within each region of interest.

### Correlation analysis between behavioral data and fMRI data

To explore further the specific function of each brain region that we found, separate correlation analyses were conducted between the behavioral data (responsibility rating scores, happiness scores and △ RT values) and the fMRI data. △ RT and the FEF activation were correlated across the three pride conditions (HP, MP and LP), r = 0.45, *p* <.005. There was also significant positive correlations between △ RT values and fusiform gyrus activation (r= 0.32, *p* <.04) and dorsal striatum activation (r= 0.39, *p* <.01) in the guilt condition. However, the subjective rating scores (guilt, pride, happiness) were not correlated with the activations of any regions across these conditions. 

**Figure 6:**
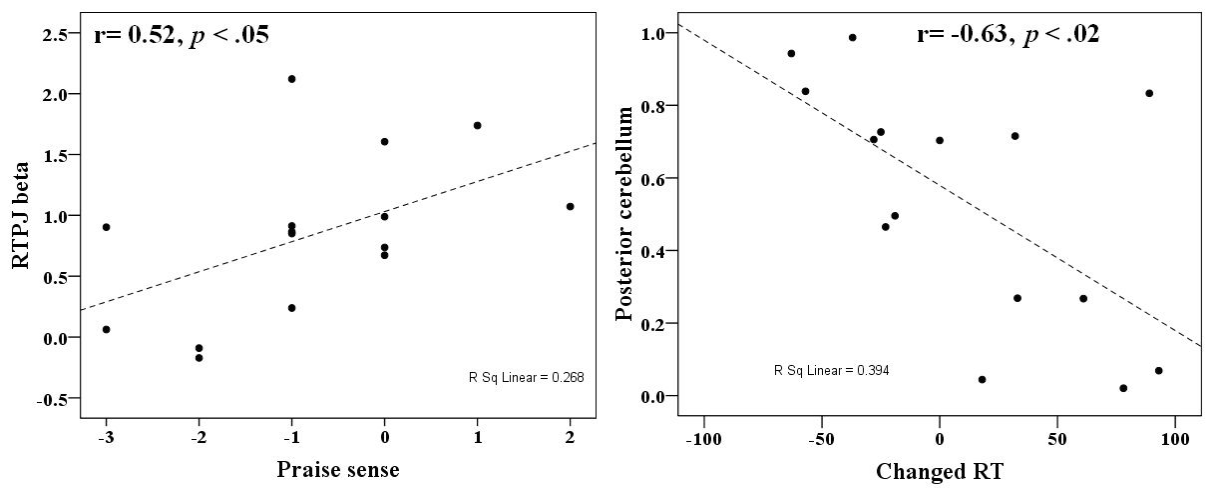
Scatter plots showing correlations between behavioral data and fMRI beta values. Left panel: the difference in subjective ratings of pride (X axis) and the difference in BOLD signal activation in RTPJ (Y axis) between the HP and MP conditions. Right panel: the difference in △ RT (X axis) and the difference in BOLD signal activation in posterior cerebellum (Y axis) between the HP and MP conditions.

Although we assumed that these fMRI results reflect responsibility attribution, it also possible that they are associated with more general cognitive functions such as performance monitoring (e.g., error detection and correction). For example, brain region activations associated with high pride might be due to the correctness of the participant’s response rather than to responsibility *per se*. To examine this possibility, we looked for brain areas sensitive to responsibility attribution by controlling for individual participant performance. Specifically, we contrasted the fMRI data associated with the HP condition with that of the MP condition, thereby equating for individual performance (because participants received positive feedback for their responses in both of these conditions; Figure 1b). Behavioral data and fMRI beta values associated with the MP condition were subtracted from those of the HP condition and the difference values submitted to a correlation analysis. The difference in pride was significantly correlated with the difference in BOLD values between the conditions for the right TPJ, r = 0.52, *p* <.05 (Figure 6): people who exhibited larger differences in right TPJ activation between the HP and MP conditions tended to report more pride in the HP condition relative to the MP condition, whereas people who had comparable activations between the HP and MP conditions reported more pride in the MP condition relative to the HP condition (Figure 6, left panel). As well, the difference in activation associated with a region in the posterior cerebellum (Declive) was negative correlated with the difference in △RT, r = -0.63, *p* <.02. 

Analogous correlations were also carried out for the guilt condition. First, difference values for behavioral data (guilt scores, happiness scores and △RT values) and for the fMRI data were obtained by subtracting these data in the MG condition from the HG condition and submitting the difference quantities to correlation analyses. No statistically significant correlations were found. 

## Discussion

The main goal of the present study was to identify brain areas underlying responsibility attribution by dissociating responsibility attribution from outcome evaluation in a decision making task. This dissociation is important because the outcome—whether the team won or lost money--is normally important to participants in such tasks, so presenting information about outcome and attribution simultaneously would confound one with the other. The behavioral results (guilt and pride responsibility scores, happiness scores and △RT values) indicated that this task did in fact arouse different degrees of responsibility sense across the conditions. Interestingly, the post-experiment debriefing did not reveal any evidence of a self-serving bias and participants even exhibited a contrary trend: they felt greater guilt when they performed poorly and their partners performed well and greater pride when both players did well. Moreover, most participants believed that team success was more important than their own performance. These results might be surprising given that the self-serving bias is commonly observed in such contexts. However, previous studies have also shown that the self-serving bias does not always occur in all experimental paradigms, suggesting that this phenomenon is not universal [34]. In the present context, the self-serving bias might not have occurred because error admission did not incur any additional costs due to the temporary nature of the team. Further, the participants involved in this study were Chinese natives, who are sometimes characterized as belonging to a collectivistic culture: our participants may have exhibited less of a self-serving bias compared to those in more individualistic societies because in China people are primarily identified as group members and are strongly motivated to behave according to group norms [38]. 

 Consistent with the behavioral data showing differential trends for reported guilt and pride (participants reported the most pride in the MP condition and the most guilt in the HG condition), the individual feedback stimuli also differentially activated several brain areas across the guilt and pride conditions. Perhaps surprisingly, more regions were activated in the pride condition relative to the guilt condition, suggesting that participants might have been relatively more sensitive to pride than to guilt. The differing valence of the reward information provided by the team feedback might have elicited differing degrees of motivation to process the individual feedback and adjust their behavior accordingly. In other words, participants may not have cared about their performance when the team lost.

 In the pride condition, several clusters of activation were found with a whole brain random effects analysis. Our behavioral data provide some insight into the functions of these regions as they relate to the present study. First, FEF activation was associated with △ RT: higher FEF activation predicted larger changes in response time following errors. Further, the FEF activations were significant stronger in the HP condition (when the partner committed the error) and LP condition (when the participant committed the error) than in the MP condition (when both team members performed correctly), but did not differ between the HP and LP conditions. These results suggest that when the participant’s partner apparently caused the team to fail, FEF activity predicted the participant’s change in RT on the following trial despite having performed the task successfully on the present trial. These finding are consistent with many previous imaging studies that show that FEF activities are responsible for attention and response selection [39]. The present study extended this finding by showing that FEF activation was not only sensitive to one’s own error but also his or her partner’s error.

 More importantly, when compared across the HP and the MP conditions, RTPJ activation was inversely correlated with participants’ pride, and in fact was the only brain region correlated with this rating. Therefore, we inferred that the RTPJ was important for social pride attribution in the present study. This finding is consistent with many previous studies that showed that RTPJ is an important region for processing ToM [40,41]. In the present study, participants worked as part of a team and thus their performance affected their partner’s results. For this reason it seems reasonable to suggest that the RTPJ might have contributed to responsibility attribution by mentalizing partner thinking about the current results. 

 This finding raises the question of the specific contribution of RTPJ to social moral processing. Young and her colleagues have found that the RTPJ is an important region for moral judgement [18,19]. More specifically, in an fMRI study, they asked participants to determine, from a third-person viewpoint, the amount of guilt attributable to different protagonists in various scenarios. They found that the RTPJ response was stronger to stories describing attempted harm by the protagonists when the protagonists believed that their action would cause harm to a second party compared to when they believed that their actions would not cause such harm, irrespective of whether the harm did or did not in fact occur [18]. Further, when RTPJ function was disrupted with TMS, participants tended to judge attempted harm as less morally reprehensible [19]. These studies demonstrated that the RTPJ not only plays a general role in belief attribution but is also sensitive to moral valence. More recently, Young and colleagues compared different ToM network activations when participants made judgements of guilt and pride. Their results showed that the RTPJ was less activated when participant judged the behavior of protagonists as praise-worthy [20]. The present results are consistent with this interpretation and extend the previous findings by showing that RTPJ also plays an important role in pride attribution from a first-person view. To be specific, the RTPJ was more strongly activated when either the participant or their partner didn’t finish the task very well despite the team winning overall. This observation suggests that RTPJ activation is not only inversely related to pride in one’s own behavior but also, in a cooperative context, to pride in a partner’s behavior. The fact that RTPJ activation did not clearly differentiate between the HP and LP conditions, in contrast to the post-experiment rating scores, might be because the RTPJ functions in a binary manner. 

 Of potential concern is that the RTPJ activation could reflect task-related motor activity because the activation pattern across conditions was similar to that of the FEF. However, previous researchers have suggested that the FEF and RTPJ belong to separate attention-related networks: FEF is located in a dorsal attention system while the RTPJ comprises one part of a ventral attention network [39]. Additionally, Mars and colleagues recently adopted diffusion-weighted imaging tractrography--based parcellation approach and identified 3 separate regions in TPJ [42]. Then the following resting-state functional connectivity analysis showed that the posterior TPJ, which is very close to the TPJ cluster found in the present study, was coupled to the areas which associated with social cognition and the default network but not the ventral attentional network. Moreover, in the present study, unlike FEF activation, RTPJ activation was not correlated with △ RT values. Furthermore, the correlation between the RTPJ activations and pride scores was conducted on the difference in brain activations and on the difference in pride ratings between the HP and MP conditions, which should control for any effects of error processing because participant performance was equated across these two conditions. Lastly, in the MP condition FEF activation was not observed whereas RTPJ was more activated relative to baseline, suggesting that these two regions might subserve different functions. 

 ACC and dorsal striatum exhibited more activation in the HP condition relative to the other two win conditions and dorsal striatum exhibited more activation in the MG condition relative to the other two lose conditions. These regions are critical for reinforcement learning [12,43-45]. Electrophysiological evidence suggests that this outcome evaluation system may operate in a binary manner, such that individual outcomes indicate that a goal has either been achieved or not [37,46,47]. In the present study, we suggest that this reinforcement learning system, especially the dorsal striatum, implemented a reinforcement learning process. On win trials, participants evidently viewed the high pride condition as the best outcome because on these trials they took personal credit for the win. By contrast, it is less clear why dorsal striatum was more activated by shared guilt on lose trials. We speculate that participants preferred to share the guilt (MG) rather than attribute all of the guilt to an individual—whether to themselves (HG) or to their partner (LG). In other words, the dorsal striatum might be responsible for the self-serving bias, consistent with the findings of a previous study [17]. Taken together, these results suggest that people view the HP condition as the optimal outcome on win trials, as reflected by the ACC and dorsal striatum activation, and the MG condition as the optimal outcome on lose trials, as reflected by the striatal activation. On the other hand, this interpretation seems inconsistent with the verbal reports of pride and guilt in these conditions: participants reported the most happiness in the MP and LG conditions. We suggest that the brain activations may reflect a relatively automatic response to the reinforcing aspects of the outcomes, whereas the verbal reports reflect a later phase of self-evaluation that may be more susceptible to culturally biased social expectations. 

Despite exhibiting different patterns of activation across the guilt and pride conditions, cerebellum and fusiform activities were both correlated with △ RT values in the different conditions: the BOLD signal of left fusiform gyrus was positively correlated with △ RT values across the three sub-conditions in the guilt condition, and the left Declive cerebellum was negatively correlated with △ RT values when these values were based on the difference between the HP and MP conditions. Even though it is difficult to determine the precise function of fusiform and cerebellum in the time estimation task adopted here, we propose that these regions contributed to processing visual and motor information for the task at hand. 

In summary, we found that RTPJ activity was associated with attributions of pride over one`s own behavior and a dACC and dorsal striatum appear to be involved in reinforcing behaviors associated with personal gain. In addition, the present study raises an interesting question: did the participants lie to the experimenter when they gave post-experiment ratings of pride? If this is true, participants may have responded relatively automatically to the reinforcing aspects of the outcomes, as revealed by the BOLD signal, whereas reported their subjective self-evaluations according to social expectations. Future studies are needed to confirm the finding that social pride attribution and self-serving bias might be mediated by different neural systems. Moreover, this study failed to find brain regions underlying guilt attribution. Thus, a novel task paradigm may be necessary to activate brain systems involved in processing feelings of guilt and pride simultaneously for a direct comparison. 
